# Targeting the Achievement Gap: Strategies Toward Removing Inequities in Undergraduate Immunology Education

**DOI:** 10.3389/fimmu.2019.02906

**Published:** 2019-12-11

**Authors:** Angelica M. Riestra, Abigail J. Morales, Frances Mercer

**Affiliations:** ^1^Department of Pediatrics, University of California, San Diego, La Jolla, CA, United States; ^2^Department of Medical Laboratory Sciences, Hunter College, New York, NY, United States; ^3^Department of Biological Sciences, California State Polytechnic University, Pomona, CA, United States

**Keywords:** URM, project-based learning, groupwork, science-identity, inflammation

## Abstract

A diverse student body enriches the classroom with lived experiences, varied skillsets, community and cultural knowledge, resiliency, and altruistic interests, all critical attributes that benefit both the classroom and the STEM field at large. However, a persistent disparity in academic and educational attainment exists between under-represented minority (URM) and non-URM students in STEM fields. This achievement gap discourages talented URM students from entering STEM professions, threatening the potential, expertise, and perspective of these professions. Here we describe the factors that contribute to the achievement gap and present strategies, utilized in our Immunology classrooms, for combating each factor. We discuss project-based learning pedagogy to give students increased agency and feelings of empowerment. We also highlight concrete practices to foster students' science identities and sense of community, factors that highly promote STEM retention. The dynamic subject of Immunology provides myriad opportunities to implement a curriculum committed to equity, as we outline below.

## Introduction

Lack of access to and discrimination in higher education has nurtured unacceptable achievement gaps between under-represented minorities (URM) and non-URM white and Asian students across STEM fields, threatening the expertise, breadth, and perspective of future STEM professions, and limiting social mobility for URMs interested in STEM (1, 2). A URM identity often intersects with First-Generation (FG) college-student status and low-income status, two other factors that can threaten student retention in higher education (3). A variety of factors contribute to the achievement gap, including stereotype threat, missed opportunities to affirm diverse values, lack of community, and a too-rigid roadmap to success. Therefore, to run equitable courses, it is imperative to minimize these roadblocks. Here, we describe practices that we have successfully utilized to break down barriers to equitable achievement in our Immunology classrooms at public Hispanic-Serving Institutions (HSIs) in New York City and Southern California.

## Stereotype Threat

The lack of diversity at the top tiers of STEM can create isolating environments for URMs and also perpetuate the insidious and inaccurate perception that non-URM white and Asian men have superior STEM abilities, thus contributing to decreased achievement of women and URMs in STEM (4, 5). Stereotype threat can be countered by highlighting the contributions of “non-stereotypical” scientists (6–8). For example, we have had success with showing diverse faces of the scientists who contributed to discovery of course content on slides (whether first author, last author, or entire labs) to portray a more accurate picture of the individuals driving the field forward. We feel it is important not to limit our “personification” of Immunology to seminal findings, but to incorporate current studies, thus leveraging the increasing diversity of the field to help offset harmful stereotypes (8). Increased representation in the classroom also helps to counter stereotype threat, as students learn from their diverse peers; thus reinforcing the importance of retaining all of our students in their Immunology coursework and in STEM curricula. Having a “non-stereotypical” instructor can trigger “stereotype inoculation” (9), demonstrating the urgency to diversify faculty; however, even a non-URM instructor can contribute to breaking down academic spaces that trigger stereotype threat by sharing personal anecdotes about content they personally struggled with, difficulties balancing academic achievement with other obligations, and strategies for overcoming these challenges. Bringing in URM guest-lecturers and encouraging students to attend seminars given by URM scientists is another tool to highlight diverse role models and minimize stereotype threat.

## Values

Many URMs that leave STEM report that they felt the majors failed to prove interesting or useful to their overall education (10). To help students affirm the value of Immunology to their lives, communities, and intended careers, we have found it crucial to incorporate a personal element into the course. This begins on the first day of class, when we invite our students to take a survey in which they reflect on why they are in the class, how taking the class fits in to their future goals, and which aspects of Immunology they care most about and why. This serves as a “utility-values” intervention, in which students personally reflect on why course material is important to them, and has been shown to foster increased motivation and to lower the achievement gap between FG-URM students and others by 61% (11). Related to this, URM students on average may be more motivated by altruistic values (12). Therefore, highlighting and giving students the opportunity to reflect on the medical or socioeconomic impact that Immunological paradigms have can be especially motivating to a group of students that is particularly interested in bridging classroom knowledge into impactful ways to support their communities (12). In parallel, giving validity to the diverse interests and insight shared by students also contributes to an inclusive classroom and helps recruit new and diverse contributors to the Immunology field. We try to mention topics or applications that students listed on their surveys as they relate to material in a lecture. Indeed, values affirmation has also been shown to decrease achievement gaps in Biology classes (13). We also use survey responses as one criterion to form groups for the final class project, so that students with similar interests can work together to develop them, as described below.

We feel that an element of self-directed and self-chosen application of Immunology class material is essential to provide space for every student in the room to affirm the value of Immunology to their particular interests and goals. This is one of the reasons why we allow students to choose the topics of their final research projects, in which they choose a disease and research the Immunological mechanisms at play during the disease. As Immunology topics vary widely from pathogen clearance to immunopathology, research projects indeed help students both master and expand course content as they delve further into mechanistic research for their presentations. Importantly, our students often choose topics relevant to themselves, their communities, and their values, such as Diabetes (disproportionately affects Latinx and Black Americans), autoimmune diseases such as Lupus erythematosus and multiple sclerosis (disproportionately affect Latinx, Asian, and African descendants), STDs (disproportionately affect Queer communities and Black Americans), and Neglected Tropical Diseases (disproportionately affect developing countries and America's poor and ethnic minorities). Self-directed learning also occurs as students work hard to master and practice utilizing their Immunological vocabulary, and as they further explore concepts to build a more robust understanding of a topic they care about. Allowing students to work on their final project in groups can increase values affirmation as described above, and also decrease grading time. We also suggest directing students to create video presentations to post on a course YouTube page, to save on precious in-class time.

## Clear and Equitable Paths to Achievement

Another factor contributing to the achievement gap is a too-rigid roadmap to success, with a perceived unnecessarily demanding pace (10). Students with more demanding lives and diverse priorities may often feel that missing one deadline or doing poorly on one exam damns them to failure in the course and may lose motivation. For this reason, we feel that having multiple opportunities for success in the class with myriad ways to earn points allows students to see a path to success even if they struggle with one particular aspect of the class. Similarly, outlining all course deadlines and milestones for success on day-1 and sticking to them, so that students can plan their work schedules, childcare arrangements, and other commitments around these dates and milestones is crucial for supporting all students equitably. Students that were not brought up in the “culture of college,” or that are not as well-networked within their Universities may also have a less clear understanding about expectations for assignments and exams. Therefore, we have found that transparency about expectations is especially critical to promote student equity. Designing clear and measurable learning outcomes is also important to provide students with a study guide for the class. In addition, posting rubrics detailing how assignments will be graded is also crucial in “lifting the veil” so that all students can easily see a clear pathway to success in the course. In some cases, providing examples of work that was previously deemed outstanding can bolster students' appreciation of their own ideas and be highly motivating. We have also found that clear and fair expectations help to establish trust of the instructor, a valuable indicator of student motivation and achievement (14).

Inclusion of low stakes, formative assessments that test in-class learning in addition to the traditional and heavier grade-weighted summative (cumulative) assessments is also beneficial for equitable achievement (15). Formative assessments are beneficial for two reasons. First, they allow students to test their knowledge, an act that has been shown to increase student learning (16). Secondly, querying student learning throughout class time also provides the instructor an avenue to gauge the level of learning in real-time and an opportunity to identify student misconceptions and clarify them on the spot. For example, when first introducing the different immune cells, a student was concerned that two cells had a similar phagocytic property. This allowed the instructor to highlight that although we categorize immune-cells into different types, two cells can express the same or similar proteins to carry out similar cell activities-highlighting the broader biological concept of structure-function. This moment also allowed the instructor to break down some of the rigidity of existing Immunology concepts (which are currently being revised with recent research findings), providing an important segue to a discussion about how we are still in the process of uncovering how different cell properties arise and currently still discovering new immune-cell functions.

## Immunology as a Foreign Language: Enhancing Language Equity

Many URM students who excel in other science curricula often find that they struggle with Immunology, as it has a daunting and complex language of its own that can be difficult to master (17, 18). Thus, many otherwise-confident URM and/or FG students experience difficulty when faced with demonstrating their knowledge of Immunology in a classroom setting or during formal examinations. In particular, English-Language-Learners (ELL) often feel that they lack the skills they need to communicate in the field's language and consequently perceive they will be ill-equipped to succeed in the course and in the Immunology field at large.

**Figure 1:**
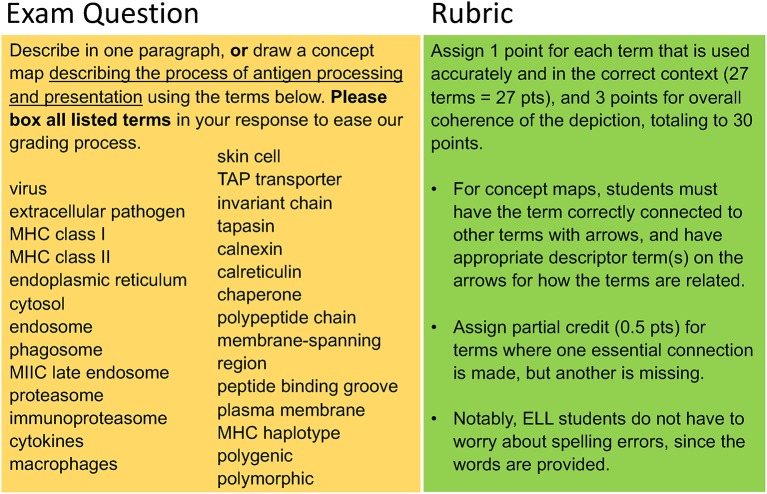
Example of exam question written with language equity in mind. An exam question is shown **(Left)** with it's grading rubric **(Right)** that enables the instructor to impartially evaluate responses that are in essay or concept map form.

There are a number of ways in which we have strived to make the language of Immunology more accessible. First, we provide students with additional resources to help them link terminology with memorable visuals and conceptual clues. One of us has found it successful to include an interactive video game, ImmuneQuest, in our curriculum, as this allows students to actively engage with the material (17). We also incorporate mnemonic devices into the lecture material to boost concept retention and memory, which is particularly useful for ELL (19). In addition, given that many students experience anxiety when confronted with essay questions on exams, we allow students to choose whether they will answer essay questions on exams in paragraph format, or by concept mapping with terms (see Stranford et al., this issue) or illustrations. To grade these questions fairly, we formulate a detailed grading rubric to accommodate both written and pictorial responses, emphasizing the accurate depiction of and connection among key terms. As example is shown in Figure 1. Indeed, encouraging students to construct and explain their knowledge through models is a powerful tool in understanding complex STEM topics and has been recognized as an authentic form of science assessment (20). The efficacy of model-building in higher education and as a practice in promoting STEM equity is currently being investigated by National Science Foundation-funded groups.

Devoting class time to “thought exercises” in which students are asked to link key concepts in Immunology has proved indispensable in building confidence among those that are apprehensive about mastering the field's technical language. Students are divided into small groups and are asked to brainstorm responses to questions that demand a connection among multiple topics in Immunology, such as “You cut yourself with a sharp knife while cooking and bacteria gets into your skin. Describe the immune response that ensues.” Thought exercises like these serve several distinct purposes. First, students are able to grasp the everyday relevance of concepts that may have previously seemed esoteric. Second, they are able to practice using Immunology's vocabulary in a non-threatening setting. Third, they are able to see how multiple concepts connect to one another. All three of these actions are critical for maximizing student learning (21) and also allow students to draw on the knowledge of their peers while simultaneously observing their own intellectual contributions to the assignment. Indeed, many studies suggest that active-learning methods enhance student performance when compared with lectures (Stranford et al., this issue) (22–24).

Additionally, we recommend ending class with an activity where students record on a piece of paper/notecard something that they have learned during that class and a “muddiest point” in which the students list something that is still unclear to them, they found difficult to understand, or on which they need additional clarification. This will allow the instructor to monitor whether they are covering the intended learning outcomes in the method and depth they intended, as well as identify areas where students experienced difficulty. In this manner, all students can seek out help and it may help to quell stereotype threat by providing an avenue for students to anonymously identify difficult concepts. By addressing these topics with the whole class, it will also reveal to the students that they were not the sole person that struggled with an aspect of the material, but rather it was a commonly challenging topic. The latter would also signal to the instructor that the topic merits further instruction time or they need to provide another instructional resource to help clarify the subject.

## Belonging, Community, and the Science Identity

Many College environments can also foster feelings of a “lack-of-belonging” for FG, transfer, veteran, and URM students, which can hinder academic success (25). Many of the active learning activities outlined in this issue (Stranford et al., this issue) help foster a sense of belonging in a learning-community as long as they are conducted in an inclusive environment. However, group work can be one of the most powerful experiences to promote a sense of belonging in college, providing students an opportunity to develop friendships and build their future professional network (26). Group work also allows students to organize their knowledge, building conceptual frameworks (21, 27). While many students bemoan group work, we have found that the following strategies promote successful group work dynamics, attested by positive student feedback. We implement purposeful group formation by giving students a survey to assess student interests and time availabilities, and also allow students to suggest peers that they would like to work with (see section Values). In addition, we ensure that groups are balanced with introverts and extroverts to aid in cohesion (28) and try to “scaffold performance” in group formation, for example not putting 2 “A” students with 2 “D” students. Group makeup is also balanced by gender and URM composition to minimize inadvertently triggering feelings of stereotype threat, and as instructors, we emphasize how the sharing diverse perspectives is an important scientific practice. To model tolerant and constructive communication, we take class-time to discuss constructive vs. destructive group work behaviors and allow students to designate the role that they will play in the group (28). Half of the grade for the group project is then assigned based on the entire group's performance and the other half is awarded to each individual in the group for the strength of their contribution and commitment to their delegated part. Students also perform “peer evaluations” of their group-members at the end of the semester, using a rubric that is posted online on the first day of class, so that students are aware of how to be good group members throughout the course. To further enhance exposure to diverse perspectives and give students increased networking chances, groups can also be “scrambled” for smaller assignments throughout the course. By facilitating productive and positive group experiences, a student's overall sense of academic and social belonging will be bolstered, contributing to student persistence and success (29).

Laboratory portions of Immunology coursework have myriad powerful virtues discussed in this issue (30, 31), including establishing a sense of community and helping students build a science identity. In working together to formulate and test hypotheses, students are able to take an active role in the scientific process. To this end, we have found great success in incorporating *true unknowns* into otherwise-controlled experiments. Allowing students to participate in generating novel data enables them to build a science identity and appreciate their increasing science-efficacy, major factors contributing to STEM retention (7, 32, 33). Additionally, mastery of techniques that are widely used in basic and clinical Immunology laboratories underscores the ongoing relevance and importance of Immunology and allows URM students to see that they have the potential to advance the Immunology field.

The last aspect of encouraging science identity is to help students envision how their gained skillsets and newfound knowledge can be utilized on their continued path to success. To achieve this, we expose students to primary literature in the classroom setting. Through reading and dissecting primary literature, students engage in higher-order thinking as they practice interpreting immunology research findings and integrate their immunology knowledge with research techniques. After discussions, one of us asks the students to formulate new hypotheses and share with the class. Powerfully, we then present examples of research laboratories and pharmaceutical companies that are pursuing similar projects to the ones students proposed. Students recognize that they are capable of thinking like scientists and future pharmaceutical company leaders. Thus, these types of exercises also affirm a student's science identity and increase science-efficacy, positively contributing to URM persistence in STEM (7, 32, 34). Importantly, students also appreciate gaps in knowledge that remain in the Immunology field and the instructor can enthusiastically highlight how students can be the ones to lead future Immunology discoveries or help deliver the latest immunological therapies. Additional in-depth discussions of using primary literature in undergraduate Immunology class (35) and focus on emerging applications (36) are included elsewhere in this issue.

## Discussion

**Figure 2:**
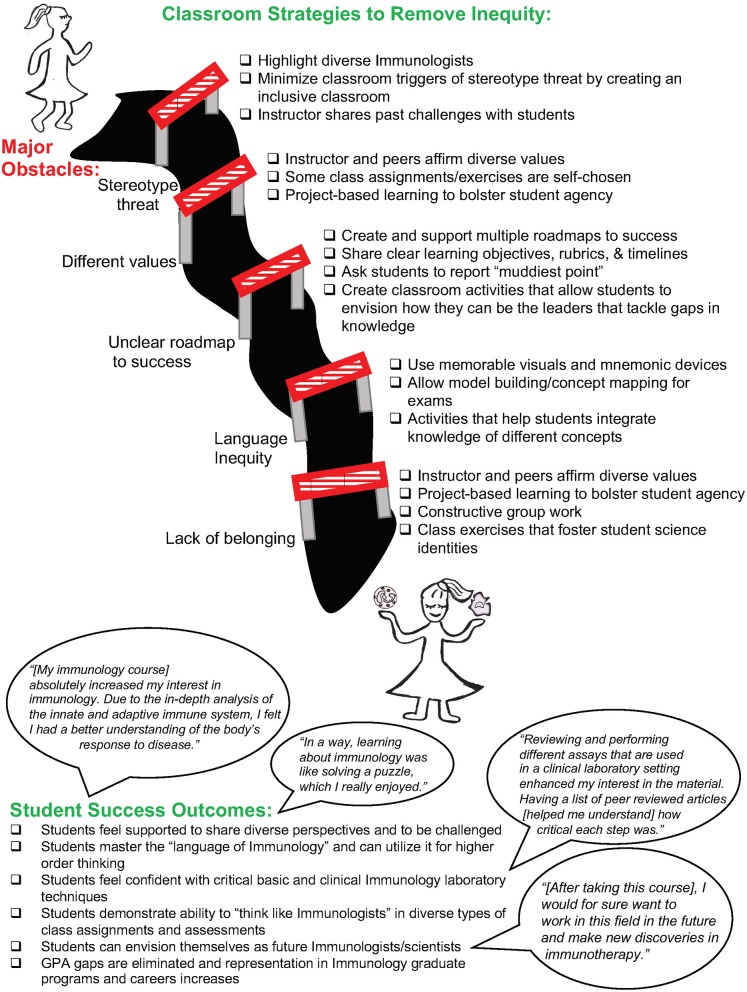
Strategies for removing barriers to student success in Immunology. This graphic outlines the major obstacles **(Left)** that prevent equitable learning in Immunology classrooms and strategies for eliminating that inequity **(Right)**. Student success outcomes, along with testimonials from undergraduates, are highlighted.

Learning communities, active learning, and other student-centered pedagogical strategies presented here, as well as creating an inclusive classroom, are all important components of the STEM persistence framework (32). Importantly, this framework helps to uplift students from *all* backgrounds, including URM, FG, low-income, and students with disabilities. Thus, a proactive approach to removing all barriers in the classroom (Figure 2) and to foster learning communities may significantly impact all students well-past their Immunology course, contributing to long-term retention in STEM and overall persistence of URMs in STEM and higher education (34, 37). Creative activities that integrate and celebrate a student's science identity, their other intersecting identities, and their diverse values are a unique contributor to URM persistence and success in STEM (38). Thus, we have presented examples of their successful implementation in our Immunology classrooms and laboratories (Figure 2). We believe that equitable teaching mirrors and leverages the diverse nature of the current Immunology field, and in turn will drive Immunology's inclusive expansion and intersections with other STEM disciplines and applications. Looking forward, we advocate for data collection on GPA gaps in specific courses so that instructors can confront and address any gaps that exist. Moreover, long-term measures of success include increased retention in the major and students pursuing advanced degrees and professions in Immunology and the biomedical sciences.

## Author's Note

The perspective presented is based on our collective classroom experiences.

## Author Contributions

AR, AM, and FM wrote and edited the manuscript.

### Conflict of Interest

The authors declare that the research was conducted in the absence of any commercial or financial relationships that could be construed as a potential conflict of interest.
